# IL-33/ST2 axis in diverse diseases: regulatory mechanisms and therapeutic potential

**DOI:** 10.3389/fimmu.2025.1533335

**Published:** 2025-01-24

**Authors:** Feiya Sheng, Mi Li, Jia-Mei Yu, Si-Yu Yang, Liang Zou, Guan-Jun Yang, Le-Le Zhang

**Affiliations:** ^1^ School of Basic Medical Sciences, Chengdu University, Chengdu, China; ^2^ College of Pharmacy, Chengdu University, Chengdu, China; ^3^ Key Laboratory of Coarse Cereal Processing, Ministry of Agriculture and Rural Affairs, Chengdu University, Chengdu, China; ^4^ State Key Laboratory for Managing Biotic and Chemical Threats to the Quality and Safety of Agro−Products, Ningbo University, Ningbo, China; ^5^ State Key Laboratory of Quality Research in Chinese Medicine, Institute of Chinese Medical Sciences, University of Macau, Macao, Macao SAR, China

**Keywords:** interleukin-33, suppression of tumorigenicity 2, central nervous system, cancer, fibrosis, autoimmune diseases, therapeutic strategies

## Abstract

Interleukin-33 (IL-33) is a nuclear factor and member of the IL-1 cytokine family. IL-33 is mainly expressed by epithelial and endothelial cells and exerts its function through interaction with various immune cells, and binding to its receptor can form the IL-33/Suppression of tumorigenicity 2 (ST2) signaling pathway. While most cytokines are actively synthesized within cells, IL-33 is produced passively in response to tissue damage or cell necrosis, indicating its role as a signaling molecule following cellular infection, stress, or trauma. IL-33/ST2 signaling pathway has been proved to play diverse role in the pathological process of central nervous system disorders, cancer, fibrosis, autoimmune diseases, etc. Although research on the IL-33/ST2 signaling pathway has deepened recently, relevant treatment strategies have been proposed, and even targeted drugs are in the preclinical stage; further research on the effect of the IL-33/ST2 signaling pathway in different diseases is still necessary, to provide a clearer understanding of the different roles of IL-33/ST2 in disease progression and to develop new drugs and treatment strategies. Because IL-33/ST2 plays an important role in the occurrence and progression of diseases, the study of therapeutic drugs targeting this pathway is also necessary. This review focused on recent studies on the positive or negative role of IL-33/ST2 in different diseases, as well as the current related drugs targeting IL-33/ST2 in the preclinical and clinical stage. The mechanism of IL-33/ST2 in different diseases and its mediating effect on different immune cells have been summarized, as well as the antibody drugs targeting IL-33 or ST2, natural compounds with a mediating effect, and small molecule substances targeting relative pathway. We aim to provide new ideas and treatment strategies for IL-33/ST2-related drugs to treat different diseases.

## 1 Introduction

Interlukin-33 (IL-33) was first identified in 1999 as a DVS27 clone in the vasospastic cerebral arteries of a canine model with subarachnoid hemorrhage (1). This cytokine is secreted by a wide range of cells, including macrophages, osteoblasts, endothelial mast cells, Dendritic cells (DCs), fibroblasts, and epithelial cells (2). IL-33 exhibits dual functions, which are manifested through its full-length form (flIL-33), acting as an intracellular gene regulator, and its mature form (mIL-33), functioning as an extracellular cytokine. flIL-33 (1-270) is abundant in endothelial cells in normal human tissues and is released to function outside cells when cells are damaged, while mature IL-33 (mIL-33) is an extracellular cytokine released by damaged cells; therefore, during inflammation, the body may counter the disease-promoting functions of IL-33 by producing mIL-33 (3). While flIL-33 is inherently biologically active and does not require maturation for suppression of tumorigenicity 2 (ST2) activation, mIL-33 is significantly more potent, exhibiting approximately ten times the activity of flIL-33 (4). This mature form is generated by the cleavage of flIL-33 by inflammatory serine proteases from neutrophils neutrophils (5) and mast cells (6), resulting in variants such as IL-33 (95-270), IL-33 (99-270), and IL-33 (109-270).

The processing of flIL-33 into its highly active mature form is crucial for its biological activity, particularly when flIL-33 is present at low concentrations or exhibits minimal activity. Initially, it was proposed that flIL-33 required processing to exhibit biological activity, leading to the belief that caspase-1 was involved in this process. However, subsequent studies have revealed that the maturation of IL-33 does not directly determine ST2 activation and that flIL-33 itself is biologically active (4). When tissue inflammation or necrosis occurs, the body rapidly releases mIL-33 into the extracellular space, where it binds to the homologous receptor ST2 on target cells, effectively activating the T helper 2 (Th2) immune response (7). Currently, research on the IL-33/ST2 pathway covers various aspects, revealing its dual role in diseases, including promoting tumor development and inhibiting tumor invasion and metastasis. Additionally, IL-33’s ability to mediate the type II immune response and induce inflammation highlights its role in maintaining various homeostatic processes.

Since the discovery of IL-33 in human tissues, extensive research has elucidated its diverse functions beyond type 2 immune responses. IL-33 has been shown to activate a wide range of immune cells associated with type 1 immunity, infections, and chronic inflammation, including Th1 cells, nature killer (NK) cells, CD8^+^ T cells, neutrophils, macrophages, B cells, and NKT cells. This versatility likely underlies the dual role of the IL-33/ST2 signaling pathway in various diseases, such as those affecting the central nervous system, cancer, fibrosis, autoimmune disorders, and inflammatory conditions. As research on the IL-33/ST2 pathway has progressed, its involvement in different diseases has become evident, necessitating a detailed analysis of the underlying mechanisms. Consequently, therapeutic strategies targeting this pathway, particularly antibody drugs, have gained significant attention. Several drugs targeting the IL-33/ST2 pathway have been developed and advanced to pre-clinical stages. Additionally, some natural compounds have shown potential in inhibiting this pathway, suggesting their future use in drug development.

Despite intensive research on drugs targeting the IL-33/ST2 pathway for various diseases, many clinical trials have been discontinued due to limited therapeutic efficacy and side effects. This review summarizes the role of the IL-33/ST2 pathway in different diseases, corresponding mechanisms, and antibody drugs that have undergone pre-clinical studies on this pathway, natural compounds and small molecules that have regulatory effects on this pathway, aiming to provide value for further understanding of drug research and mechanisms in the future.

## 2 IL-33/ST2 signaling pathway

As a nuclear factor, IL-33 mainly binds to chromatin in nucleus to regulate the expression of inflammatory factors and other responses. IL-33 initiates its signaling pathway by binding to the ST2 receptor, consisting of interleukin 1 receptor-like 1 (IL1RL1) and coreceptor coding, and binds specifically to IL-33. ST2 is a member of the Toll-like/IL-1 receptor superfamily, encoded by IL1RL1 (8). There are two main subtypes of ST2: Transmembrane ST2-ST2L, which has a transmembrane structure, including transmembrane segments and Toll/IL-1TR receptor intracellular domains (4); Soluble ST2 (sST2), no transmembrane structure, can be secreted into the extracellular (9). The binding of IL-33 to the receptor ST2 involves conformational changes. The binding of the C-terminal IL-1-like cytokine domain of IL-33 to the ST2L receptor induces conformational changes and the recruitment of IL-1 receptor helper protein (IL-1RAcP), leading to the formation of a heterodimer receptor complex on cell membrane, and IL-33 is inactivated after maturation by caspase-1 (10). The heterodimerization connects the intracellular domains of the two transmembrane proteins, stimulating the recruitment of junction molecules and mediating the transduction of the IL-33 signal through these molecules.

Distinct from other cytokines, IL-33’s nuclear localization (nIL-33) and regulation set it apart. In the absence of stimulation, nIL-33 localizes to the nucleus due to its nuclear localization signal and undergoes various post-translational modifications, including SUMOylation, acetylation, and ubiquitination (11). SUMOylation, involving the attachment of a small ubiquitin-like modifier protein (SUMO) to the target protein, has four potential sites identified within IL-33, promoting T cell proliferation (12). Acetylation, mediated by Histone acetyltransferase (KAT8), plays a significant role in allergic inflammation (13). Ubiquitination targets IL-33 for post-translational modification, with the deubiquitinating enzyme USP21 interacting with IL-33 and localizing it within cells (14). Investigating these post-translational modifications’ regulatory mechanisms may reveal potential therapeutic targets for various diseases. For example, lactylation (15), a novel post-translational modifier, is crucial in neuroregulation, endotheliostromal transformation, tumor transformation, and the tumor microenvironment (16). Additionally, Meng et al. (17) proposed two lysine propionylation methods to predict the localization and functional regulation of nIL-33 via propionylation of IL-33, although further validation is required.

The dual-primers subsystem drives the differential expression of mRNA to produce ST2L and sST2. ST2 receptor is primarily expressed by the immune cells that participate in innate immune response, including mast cells, lymphoid cell type 2 (ILC2), macrophages, eosinophils, basophils, and natural killer (NK) cells (18). Therefore, the purpose of ST2 expression is achieved through adaptive immune cells, such as CD4^+^ T cells, CD8^+^T cells, and T regulatory cells (Tregs) (19). In addition, IL-33 can also affect the immune response of the body by activating other subpopulations related with type 2 immunity and allergic inflammation, such as Th2 cells, basophil cells, eosinophilic cells, M2 macrophages, and DCs, to cooperate with ST2 and exert the immune role of this pathway (20).

## 3 IL-33/ST2 axis in allergic cells

**Figure 1:**
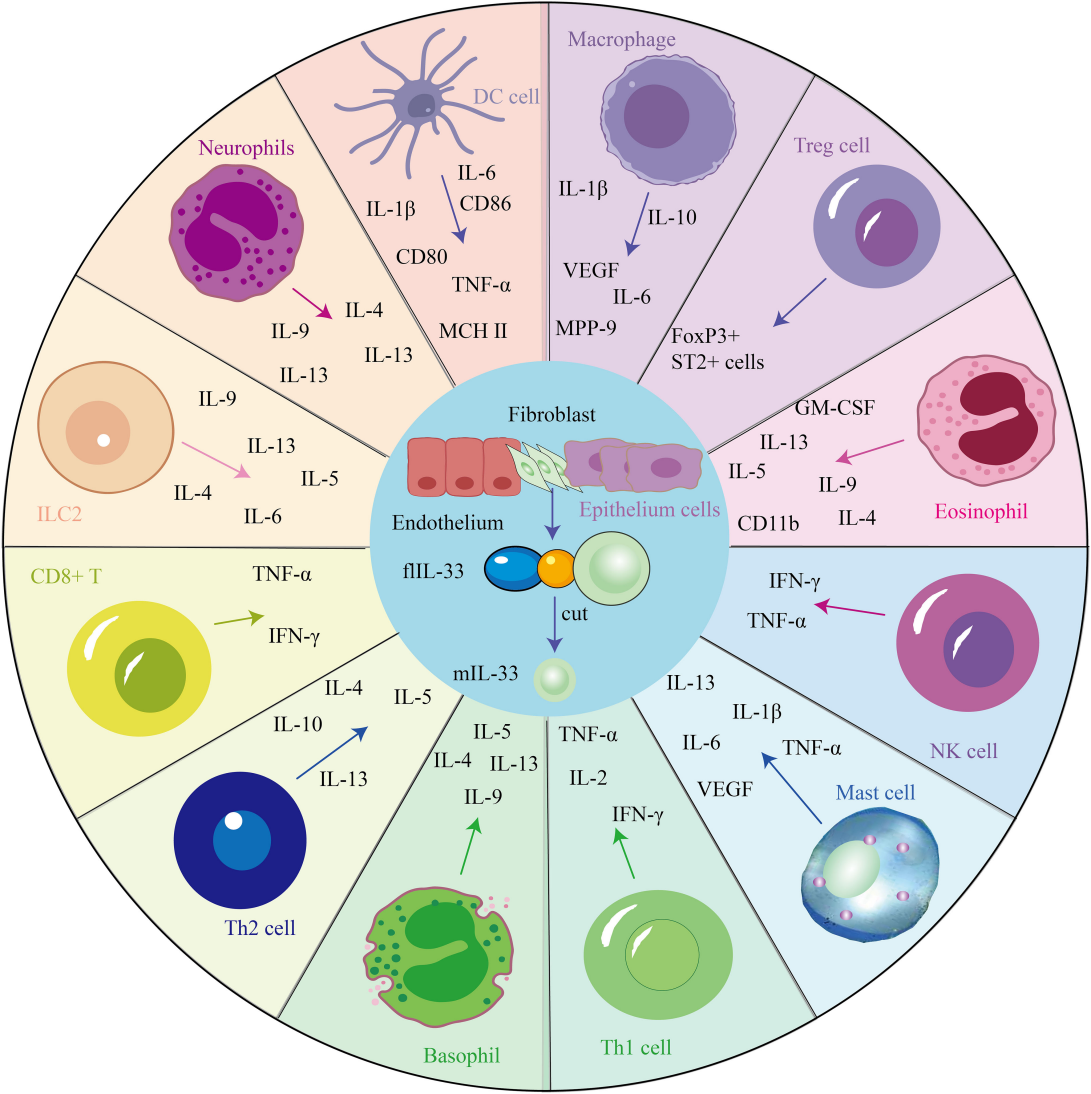
IL-33 mediates the secretion of cytokines via several cells and is involved in several disease processes.

IL-33 could act as an inflammation moderator. The IL-33/ST2 signaling pathway plays a pathological and protective role in many diseases and is involved in regulating inflammation and tissue repair and the balance between the immune response, as several immune and non-immune cells express the ST2 receptor (21), such as mast cells, T cells, eosinophils, macrophages and other cells (**Figure 1**). Therefore, IL-33 blockade may be a new treatment for allergic diseases, and some promising leading compounds (blockers) have recently been discovered. The following is a summary of immune cells mediated by the IL-33/ST2 pathway, which plays an important role in maintaining body homeostasis.

### 3.1 IL-33/ST2 axis and mast cells

Mast cells have been identified as critical mediators of inflammatory diseases. A recent study has shown that infiltrating mast cells can stimulate ICOS regulatory T cells via IL-33 in tumors (22). IL-33 is a strong initiator of type 2 immune responses and an activator of mast cells. IL-33 induces the production of inflammatory mediators in mast cells in a nuclear factor kappa-B (NF-κB) and mitogen-activated protein kinase (MAPK)-related manner (23). Further injecting IL-33 into mice leads to the progression of dermatitis, which depends on the activation of dermal mast cells by ST2 (24). IL-33 activates mast cells by binding to ST2, and inflammatory proteases are released by mast cells and act on the body’s immune response. In addition, mast cells release cytokines, such as TNF-α, IL-1β, IL-6, IL-13, and vascular endothelial growth factor (VEGF), with immunoglobulin E (IgE) and non-IgE stimuli.

### 3.2 IL-33/ST2 axis and T cells

ST2 is first expressed on the differentiated Th2-type CD4+ T cells (25). In addition, IL-33 is a chemoattractant of Th2 cells, as an adaptation to transfer Th2 cells to mice without ST2 and then injecting IL-33 into the footpad results in the accumulation of metastasized Th2 cells in tissues (26). In addition, IL-33 directly induces polarization of naïve T cells to the Th2 phenotype by activating mouse DCs to participate in Th2-mediated immune responses (27). In addition to interacting with Th1/Th2, IL-33 reduces the differentiation of T cells into Th17 cells *in vivo* and *in vitro* (28). In addition, the IL-33/ST2 axis in Tregs increases the frequency of Tregs and decreases the production of IL-17 and interferon-γ (IFN-γ) in disease (29). IL-33 induced the expansion of Th2 cells and enhanced the proliferation of ST2 and Tregs in epithelial-derived IL-33.

### 3.3 IL-33/ST2 axis and eosinophils

IL-33 not only induces the production of eosinophils in mouse models (30) but also increases the survival rate of eosinophils. ST2 mRNA and protein can be detected in eosinophils, and anti-ST2 inhibits eosinophils’ response to IL-33; therefore, IL-33 and its receptor, ST2, can be therapeutic targets for mediating diseases caused by eosinophils. However, the role of IL-33 in degranulating human eosinophils is controversial: IL-33 does not induce degranulation (31). However, other studies have shown that it induces degranulation assessed by releasing neurotoxins derived from eosinophil-derived neurotoxins (32). In addition, IL-33 can achieve significant release by stimulating the C-C motif of the pro-inflammatory cytokine IL-6 and chemokines IL-8, also called C-X-C Motif Chemokine Ligand 8 and C-C motif chemokine ligand 2 in eosinophils (33). IL-33 is a potential activator of the innate immune system. For example, injecting IL-33 into mice induces increased eosinophilia production, superoxide anion, and IL-8 and promotes cell survival. Intriguingly, a recent study has shown that IL-33 can enhance the anticancer activity of eosinophils by reprogramming tumor cells, suggesting a novel approach for cancer therapy (34).

### 3.4 IL-33/ST2 axis and macrophage

IL-33 is also expressed in hematopoietic cells, particularly in macrophages and DCs. IL-33 is present in hematopoietic-derived cells, particularly macrophages and DCs, and in endothelial and epithelial cells of the barrier tissues (35). In addition, anti-inflammatory cellular proteins downregulate IL-33-induced macrophage activation (36). Macrophages are very important cells in producing molecules that affect nasal receptor neurons, causing hyperalgesia. They can modulate IL-33 or target IL-33. Macrophages mainly have two polarization states, namely M1 and M2. IL-33/ST2 signaling pathway mainly regulates the M2 polarization of macrophages, and M2 macrophages can mediate the secretion of various cytokines, such as IL-1β, IL-6, IL-8, granulocyte-macrophage colony-stimulating factor (GM-CSF), and VEGF. In the tumor environment, these factors promote tumor angiogenesis, invasion, metastasis, and immune escape; therefore, targeting macrophages against the IL-33/ST2 pathway also becomes a potential therapeutic strategy.

### 3.5 IL-33/ST2 and other cells

The IL-33/ST2 axis has been documented to be involved in local cellular responses in fibroblasts, epithelial cells, and endothelial cells. For example, the IL-33/ST2 axis can lead to depositing extracellular matrix (ECM) components in the airway (37). In addition, IL-33 stimulates dermal fibroblasts to release various chemokines through a process dependent on extracellular regulatory protein kinase (ERK) and p38 MAPK pathway activation, thereby inducing eosinophilic infiltration. Eosinophils secrete various inflammatory factors, such as IL-4, IL-5, IL-9, IL-13, and GM-CSF, and are involved in promoting and inhibiting various diseases. In a 4T1 bone cancer pain model, IL-33 expression was observed in astrocytes but not in neurons and microglia. Earlier studies have shown that IL-33 in the spinal cord causes neuropathic pain and that mechanical hyperalgesia caused by chronic systolic injury is reduced in mice lacking ST2. In addition, ST2 deficiency led to reduced activation of spinal cord astrocytes and microglia, confirming a reduction in neuropathic pain (38). In the spinal cord contusion model, IL-33 is mainly expressed by astrocytes rather than microglia. In experimental autoimmune encephalomyelitis models that mimic demyelinating diseases of the central nervous system (CNS), such as multiple sclerosis (MS), spinal cord neurons express both IL-33 and ST2, while astrocytes only express IL-33 (29). Therefore, the IL-33/ST2 axis may be required in glial cells and may vary from disease to disease; therefore, detecting IL-33 and ST2 expression in different diseases may be helpful in the diagnosis and treatment of diseases. In addition, IL-33 expression activates B cells to activate delayed-type hypersensitivity in a mouse model. IL-33 also amplifies IL-4R-expressing B cells and acts as an antigen-related stimulus of IgE synthesis in mouse B cells. Activated IL-33 can act on ILC2 and drive the proliferation and recruitment of ILC2, and ILC2 can produce various cytokines, including IL-6, IL-4, IL-5, IL-9, and IL-13, which further promote Th2 differentiation and participate in the development of various diseases (39).

## 4 IL-33/ST2 axis in diverse diseases

**Figure 2:**
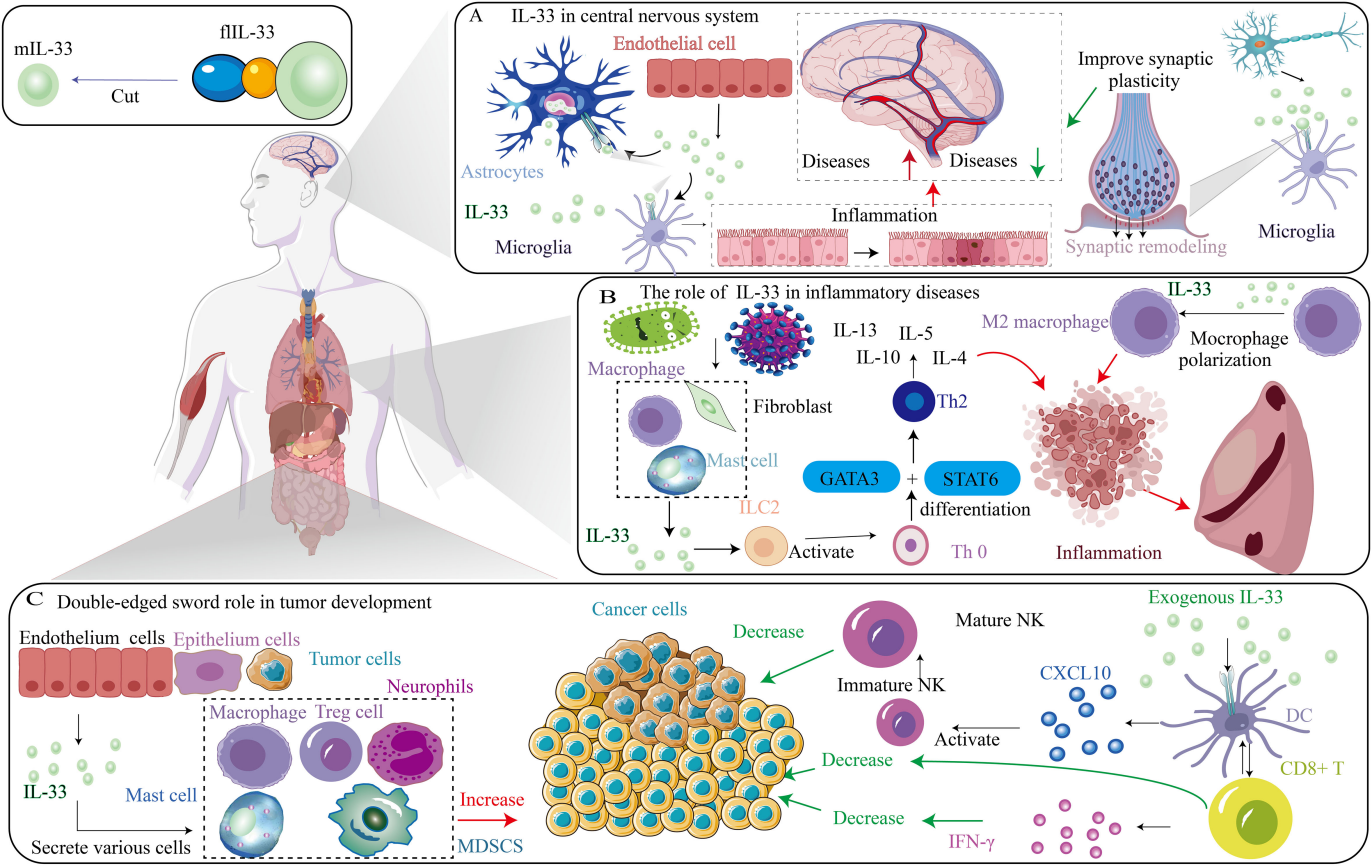
Different role of IL-33/ST2 axis in diverse diseases. **(A)** The role of IL-33 in central nervous system. **(B)** The role of IL-33 in inflammatory diseases. **(C)** Double-edged sword role of IL-33 in tumor development.

Emerging studies have proved that the IL-33/ST2 axis is involved in diverse immune responses, restoring normal tissue homeostasis via promotion of wound healing and repair, and is essential in tumor progression. The IL-33/ST2 axis induces necrosis from damage to stromal cells and releases flIL-33, which activates the heterodimer ST2L/IL1-RAcP complex for signaling through the Toll/interleukin-1 receptor (TIR) domain of IL-1RAcP. Moreover, intensive studies have shown that the IL-33/ST2 axis is involved in the inflammatory response related to Th2 and activates tissue remodeling. IL-33/ST2 signaling can promote cell-dependent inflammatory responses and may have opposite effects in inflammatory or allergic conditions or diseases (such as cancer and autoimmune diseases) (40), reflecting the duality of action of the IL-33/ST2 pathway. Therefore, the roles of the IL-33/ST2 signaling pathway in diverse systems differ (**Figure 2**).

### 4.1 CNS diseases

The interplay between the immune system and the central nervous system (CNS) is vital for maintaining CNS homeostasis and contributing to the development of CNS diseases. Recent extensive research has highlighted IL-33 as a key cytokine that facilitates this intricate neuroimmune communication. Studies have demonstrated that IL-33 plays an essential role in both CNS development and homeostasis (41). This cytokine is expressed at the mRNA and protein levels in various immune-related cells, including endothelial cells, some epithelial cells, and mast cells (42). Notably, IL-33 has been implicated in neuroinflammation across a range of neurological disorders, including Alzheimer’s disease (AD), Parkinson’s disease (PD), multiple sclerosis (MS), schizophrenia, and central nervous system injury (CNSi).

IL-33 is synthesized by endothelial cells and astrocytes, while microglia or neurons do not contribute to its production. The expression of IL-33 receptors is predominantly observed in microglia and astrocytes. Using a dose-dependent method, increased synthesis of IL-33 microglia leads to the production of IL-1β, such as TNF-α and IL-10 anti-inflammatory cytokines. Additionally, IL-33 strengthens the secretion of chemical factors, production of nitric oxide, and cellular activity inside the microglia. Ultimately, CNS IL-33 activates microglial cells, potentially acting as a vector of pro-inflammation in CNS pathophysiology. Microglia cells are the primary immune cells in the brain parenchyma, regulating the pattern of neural circuits during early development, and are vital in regulating brain homeostasis throughout life. Double-labeled immunofluorescence staining demonstrated that IL-33 and ST2L are expressed primarily in astrocytes and microglia. Moreover, ST2 expression in the brain mirrors that of IL-33 regarding distribution pattern.

The role of IL-33/ST2-AK2-dependent metabolic adaptation in promoting microglia phagocytosis during early brain development implicates neurodevelopment and neuropsychiatric disorders. Similarly, disrupting the IL-33/ST2-AK2 signaling pathway inhibits microglia metabolic adaptation and phagocytosis, causing impaired neurodevelopment and neurological disorders (43). Additionally, the microglia-astrocyte circuit plays an important role in the normal development of nerves and CNS diseases. However, the role of the IL-33/ST2 axis in the CNS needs to be further studied.

#### 4.1.1 Neurodegenerative disorders

Neurodegenerative diseases (NDs) are primarily sporadic and genetic disorders of the CNS characterized by a gradual decline in the function of neurons and their connections in a specific population. These diseases exhibit shared pathological traits, including mitochondrial dysfunction, oxidative stress, and inflammatory responses to excitatory toxins. NDs encompass conditions such as AD, PD, and MS. Many studies within 10 years on the onset of degenerative disease care mechanism focused on eight traits of NDs: pathology protein accumulation, neural synapse dysfunction, harmful protein function, the cytoplasm, energy immutable ideal of routine changes, DNA and RNA, and inflammation neuron death. In addition, they also submitted various customized treatment measures, providing a new direction for future clinical translation (44).

The mechanisms by which IL-33 boosts stable synaptic remodeling through microglia suggests that IL-33 stimulation of microglial phagocytes promotes increased synaptic elasticity (45). The expression level of IL-33 may vary depending on the different diseases, cell types, or sample sources. However, a study has shown that IL-33 plays a protective role in CNS cells. Elevated expression of IL-33 in oligodendrocytes (OLs) protects OLs from oxidative stress by maintaining myelin and OLs homeostasis during demyelination (46). IL-33 expression levels may differ from other pathogeneses, depending on the neurodegenerative subtype. However, some evident commonalities also exist between the various NDs. The exact physiological activity of the IL-33/ST2 signaling pathway in NDs is unknown. IL-33 may be a promising target for pre-labeled and NDs therapeutic targets (47).

##### 4.1.1.1 AD

AD is the prevalent form of dementia in the elderly, impacting cognition, memory, independence, and quality of life, ultimately leading to death (48). A hallmark of AD is the presence of amyloid protein, derived from amyloid precursor protein (APP), which normally aids neuron growth and repair. APP is a protein that undergoes cleavage by A-secretase and gamma-secretase for breakdown and recycling. However, under pathological conditions, β-secretase and γ-secretase cleave APP, producing insoluble fragments that form beta-amyloid monomers. These sticky monomers aggregate into amyloid beta plaques on neurons, disrupting signal transmission and impairing brain functions like memory. These plaques also elicit an immune response and inflammation, further damaging nearby neurons. IL-33 is constitutively expressed in CNS and plays a crucial role in neuropathological injury response in glial cells (49). Under normal circumstances, neurons, microglia, astrocytes, and oligodendrocytes all express IL-33, while ST2 is mainly expressed by microglia, neurons, oligodendrocytes, and astrocytes. However, the level of IL-33 is associated with cognitive protection in patients with AD (50). Neuronal damage and degeneration can stimulate the release of IL-33 from glial and mast cells. Once released, IL-33 promotes microglia proliferation and the expression of pro-inflammatory factors such as TNF-α and IL-1β, as well as the anti-inflammatory factor IL-10 (51).

The dual effect of ST2 as a receptor for IL-33 exists in two primary forms, ST2L and sST2. When IL-33 binds to ST2L, it triggers a downstream signaling pathway for clearing amyloid plaques, leading to a therapeutic effect on AD (52). In contrast, sST2 competitively binds to IL-33 and mitigates neuroprotective effects, and studies have shown that high levels of sST2 demonstrated mild cognitive impairment and AD. Additionally, the study shows that plasma sST2 increases NDs and is associated with worse cognition. Higher levels of sST2 serve as a potential biomarker of the disease in neurodegeneration. Several ideas reveal the potential of the IL-33/ST2 pathway for detecting new biomarkers and therapeutic targets in various brain diseases (53).

An early study suggested that IL-33 plays an important role in neuronal repair during aging. Mice without IL-33 develop AD, which is characterized by tau abnormalities and significant neuronal loss in the hippocampus and accelerated aging, memory or cognitive impairment, and cerebral cortex. In IL-33-deficient mice, neurons exhibit rapid accumulation of abnormal tau protein, DNA double-strand breaks, and abnormal autophagy vesicles (54). In animal models of AD, cytokine IL-33 enhances the phagocytosis of microglia against Aβ while reducing their pro-inflammatory response (55). In addition, IL-33 polarizes macrophages or microglia into an anti-inflammatory phenotype and decreases levels of pro-inflammatory factors such as IL-1β and IL-6. Enhanced microglial phagocytosis activity improves clinical symptoms while reducing soluble Aβ levels and plaque formation.

##### 4.1.1.2 MS

MS is a neurodegenerative disease characterized by demyelination, inflammation, and glial scarring in multiple regions of the white matter of the CNS (56). MS significantly impacts the daily lives of individuals through sexual dysfunction, orthostatic dysbiosis, gastrointestinal symptoms, and neurological disorders. IL-33 expression levels are elevated in patients with MS, with increased levels observed in the brain lesions of these patients (57). Studies have shown that IL-33 protein is expressed by various types of resident cells of the CNS (58), such as neurons, astrocytes, microglia, and oligodendrocytes, while ST2 is mainly expressed by neurons and oligodendrocytes. Therefore, IL-33 serum may be an important biomarker.

IL-33 knockout of activated astrocytes and microglia in the experimental autoimmune encephalomyelitis mice showed a protective effect of IL-33, with increased production of TNF-α in primary microglia and astrocytes (59). However, the direct effect of IL-33 on myelination and its underlying mechanisms still remains to be further clarified. In addition, the progression of the IL-33/ST2 signaling pathway in MS is its expression and inhibition of myelination in the CNS through oligodendrocytes. The cell expression profile of IL-33 and ST2 was mapped in the brain tissues of patients with MS and corresponding controls via immunohistochemistry, and the results indicate that IL-33 may play a crucial role in MS occurrence and development by inhibiting myelination and oligodendrocyte expression in the CNS (60). However, its exact function remains to be further determined before potential treatment strategies can be developed.

##### 4.1.1.3 PD

PD is a prevalent neurodegenerative disorder affecting older adults, with an average onset age of 60 years and a prevalence of about 1.7% in those over 65. PD’s primary pathology involves degeneration and death of substantia nigra dopaminergic neurons, leading to markedly reduced striatal dopamine levels (61). The exact cause remains unknown, but genetic, aging, environmental, and stress factors may contribute. Patients with PD exhibit significantly higher serum IL-33 concentrations and upregulated IL-33 expression in the brain (striatum and midbrain) compared to healthy individuals (62). Mast cells, expressing the IL-33 receptor, are activated in PD brains and may drive neuroinflammation. Glial cells and neurons interact with mast cells to exacerbate neuroinflammation, representing a novel therapeutic target for PD. In mice, mast cell protease-6/7 (mMCP-6/7) activates glial cells and astrocytes to release IL-33, and selective IL-33 activation in mast cells induces astrocyte activation and upregulation of p38 and NF-κB, key markers of pro-inflammatory cytokines (63).

#### 4.1.2 Schizophrenia

Due to the complex pathogenesis of mental diseases, attention to them has recently surged. Multiple potential causes and targets can alter developmental trajectories and mental functions, manifesting differently phenomenologically. Changes in brain structure and neurodevelopmental outcomes, influenced at various tissue levels, complicate the diagnosis and treatment of psychiatric disorders. Studies indicate that autoimmune responses and IL-33 axis disturbances may play a role. Schizophrenia, a complex neuropsychiatric disorder featuring hallucinations and delusions, shows an association between IL-33 levels and disease progression. Serum IL-33 positively correlates with cognitive performance in schizophrenia patients, and elevated serum IL-33 and sST2 levels are linked to improved cognition, whereas IL-33-deficient mice show behavioral deficits (64). In addition, increased levels of inflammatory factors, including IFN-γ, IL-1β, IL-12, and TNF-α, have been found in the brains and blood of schizophrenia patients (65). In addition, the IL-33 gene polymorphism (rs11792633) has been documented to be associated with the development of schizophrenia (66). Various exact functions remain to be explored in the future development of schizophrenia-related drugs.

#### 4.1.3 CNSi

CNSi is mainly characterized by Type 1 or 2 immune responses and has a great impact on CNS repair and damage recovery process (67). Therefore, IL-33, an early warning factor released by damaged cells and essential immunomodulatory cytokine, plays a crucial role in CNSi pathophysiology (68). IL-33 plays an essential role as a marker in many diseases. For example, increased serum sST2 levels in patients with stroke are associated with worsening symptoms. Serum IL-33 levels are elevated in patients with acute ischemic stroke (AIS) and have been used as a diagnostic and prognostic biomarker for AIS (69). IL-33 achieves neuroprotective effects in mouse models by inhibiting inflammation, apoptosis, and autophagy pathways, such as cerebral hemorrhage, and by exerting neuroprotective effects through anti-inflammatory pathways. In addition, activating IL-33/ST2 signaling in an ischemic brain improves astrocyte response in neonatal hypoxic-ischemic brain injury, thereby protecting ischemic neurons in a glia-derived neurotrophic factor-dependent manner (70). In addition, the IL-33/ST2 signaling protects against traumatic brain injury by enhancing Treg function (71). These studies suggest that the IL-33/ST2 axis represents an important target for disease treatment. IL-33/ST2 signaling pathway mediates acute inflammatory, neuropathic, and chronic pains through the action of nerve cells, and the nerve cells involved in the occurrence and maintenance of chronic pain are mainly activated astrocytes.

### 4.2 Cancer

As cancer research advances, the IL-33/ST2 signaling pathway’s role in cancer has gained attention. Evidence shows that IL-33/ST2 activation can both promote tumor development, regulating aspects like angiogenesis, invasiveness, metastasis, and immune protection, and exhibit anti-tumor effects, aiding tumor regression (72). This pathway is implicated in the inflammatory tumor microenvironment (TME), potentially serving as an early diagnosis and prognosis marker. IL-33/ST2 regulates immune cells in the TME through multiple intracellular kinases and factors. IL-33 is involved in various cancers. Multiple studies have proved that IL-33/ST2 signaling pathway is involved in inflammatory TME and may be a marker for early-stage diagnosis and prognostication of tumors. Therefore, targeting the IL-33/ST2 axis is a promising strategy for cancer immunotherapy.

#### 4.2.1 Regulatory role of IL-33 in the TME

Tumor development primarily results from mutation accumulation, but non-tumor cells and molecules also contribute to malignancy by shaping the tumor microenvironment (TME) (72). The TME is crucial in tumorigenesis, regulating proliferation, invasion, and metastasis. The IL-33/ST2 axis modulates the TME effectively, influencing tumor progression through target cells and downstream pathways activated by tumor- or non-tumor-derived IL-33 (73). IL-33 recruits immune cells that secrete molecules impacting tumor phenotype, including macrophages, mast cells, MDSCs, Tregs, and neutrophils, fostering a tumor-promoting environment and a self-sustaining pro-inflammatory loop (74).

Despite conflicting reports on IL-33’s pro- or anti-tumor effects, its role as a potent TME regulator is clear, influencing tumor phenotype and malignancy via immune cell recruitment. IL-33 affects Tregs, promoting tumor immune escape, and CD8+ T cell-derived IL-33 mediates cytotoxicity within the TME. The IL-33/ST2 signaling pathway plays a dual, environment-dependent role in tumor development, modulating tumor-related immunity by remodeling the TME to either promote immunosuppressive cell recruitment or inhibit tumor growth by enriching NK and cytotoxic T cells.

**Figure 3:**
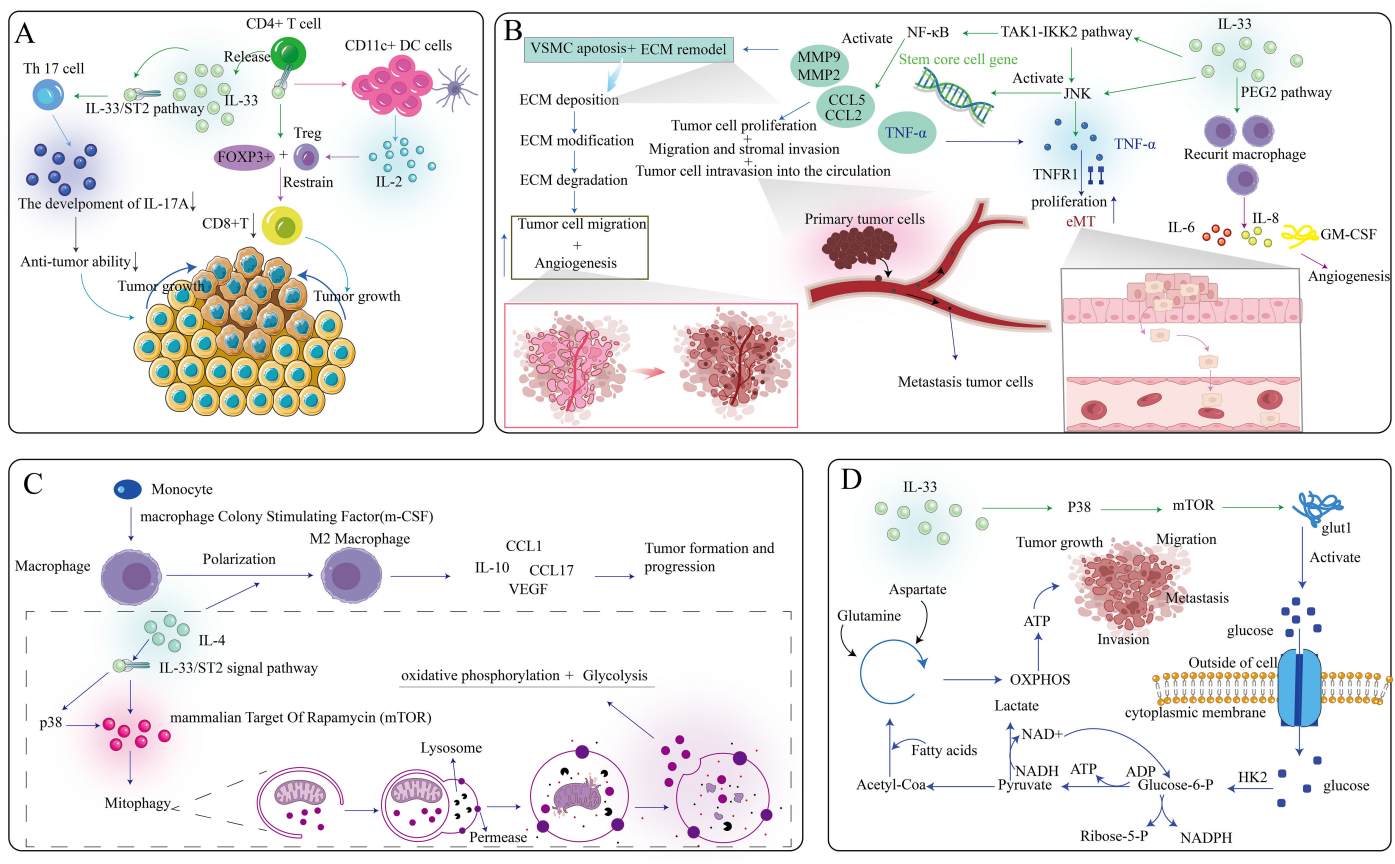
Multifaceted role of the IL-33/ST2 axis within the tumor microenvironment. **(A)** IL-33, secreted by CD4+ T cells, specifically targets Th17 cells and CD11c+ DC cells, ultimately contributing to the diminution of anti-tumor effects and the promotion of tumor growth. **(B)** The IL-33/ST2 axis exerts its influence on multiple pathways, thereby modulating tumor cell invasion and angiogenesis. **(C)** The IL-33/ST2 axis affects the polarization of macrophages, further facilitating the growth of tumor cells. **(D)** IL-33 can impact the release of intracellular ATP, subsequently influencing the growth, migration, invasion, and metastasis of tumor cells.

Immune surveillance, a key immune system function, involves recognizing and eliminating mutated cells to prevent tumorigenesis. Type 1 immune response is a key component of cell-mediated anticancer immunity and is characterized by tumor-induced IFN-γ-producing Th1 cells, NK cells, NKT cells, cytotoxic T lymphocytes, and γδ T cells (75). NK cells can stimulate cDC1 into TME to promote cancer immunogenicity (76). However, in cancer patients and tumor-bearing mice, many tumor-infiltrating Th1 and CD8+ T cells are unresponsive due to immunosuppressive mechanisms (77). The IL-33/ST2 signaling pathway is a potent TME regulator, recruiting immune cell populations that reshape the TME to either promote malignancy or induce tumor regression, particularly those related to type 2 and regulatory immune responses (78). Cytokine regulation is vital in tumor development, directly affecting tumor immunogenicity and tumorigenesis. Reduced IL-33 expression in tumor cells decreases immunogenicity, while stromal IL-33 promotes progression and metastasis by mobilizing MDSCs and Tregs. Evaluating anti-IL-33 antibodies for their anti-cancer effects is worth considering. The pro-tumor effect of IL-33/ST2 signaling in the TME depends on target cells and signaling pathways involved, summarized in **Figure 3**.

#### 4.2.2 IL-33/ST2 axis affects tumor growth

A type of tumor cell with long-term tumorigenic ability, known as tumor-initiating cells (TIC), is crucial in cancer development and the study of therapeutic resistance. However, the study of effective TIC-targeted therapies is inadequate because of low sensitivity to identifying TIC vulnerability. TIC is critical in creating a niche microenvironment for tumor progression and acquired drug resistance. The antioxidant activity of TIC is mediated by the transcription factor nuclear factor related factor 2, which promotes the release of IL-33. This cytokine facilitates the differentiation of macrophages expressing the high-affinity IgE receptor FceRIa, which is close to TIC. These IL-33 responsive FceRIa+ macrophages send para secretory transforming growth factor β (TGF-β) signals to TIC, inducing aggressiveness and drug resistance and further upregulating IL-33 expression (79). This TIC-driven IL-33-TGF-β feedforward loop exhibited potential in cancer treatment.

In most TMEs, macrophages are the main stromal component. Macrophages are an essential part of the TME. M1 macrophages secrete various pro-inflammatory factors that suppress tumor growth and development, while tumor-associated macrophages (TMS) mainly exhibit M2 phenotype. Regarding mechanism, IL-33 activates the p38- GATA binding protein 3 (GATA3) signaling pathway to induce M2 polarization of macrophages, promote Th2 cells and ILC2 to secrete tumor-promoting cytokines, mainly IL-4, IL-5, and IL-13, thereby playing a role in promoting tumor development (80). Studies have shown that macrophages have an impact on tumor progression, and macrophages promote tumor growth. Targeting this pathway, a recent study has published a combination of IL-33 and p38 inhibitors, which synergistically inhibit tumor growth and metastasis (81). IL-33 can regulate the mammalian target of rapamycin through the p38 MAPK pathway to induce facilitative glucose transporter 1, activate the uptake of glucose by cells, and complete oxidative phosphorylation in cells to obtain ATP, providing energy for the migration, invasion, and development of tumor cells (82). In addition, a study (83) has shown that immunosuppressive MDSCs can promote tumor development by stimulating the autosecretion of GM-CSF to form a cumulative positive feedback loop. IL-33 mainly mediates the activation of Jun N-terminal kinase (JNK) and NF-κB through MAPK kinase 7 (MAP3K7, also known as TAK1). Through the TAK1-inhibitor of the kappa B kinase 2 pathway, IL-33 can also induce cancer cell stem cells by activating the JNK or NF-κB pathway, thereby activating the development process of tumor cells (84). JNK can also induce the secretion of TNF-α, play a role in combination with TNF receptor 1, and promote the role of Epithelial-Mesenchymal Transition (EMT) in the tumor environment to promote tumor proliferation (85).

In addition, IL-33 can affect the infiltration of various immune cells into the TME to affect the tumor process. IL-33 promotes tumor growth and increases the infiltration of ST2^+^ Treg cells. In addition, ST2^+^ Tregs from tumor tissue inhibit CD4^+^ and CD25 T cell proliferation and IFN-γ production, promoting tumor progression. The binding of the ST2 receptor on CD4+T cells to IL-33 induces Forkhead box protein P3^+^ (FOXP3+). Treg inhibits the action of CD8+T cells, thereby promoting tumor progression. In a colon cancer study, deleting the ST2 gene in mice led to the infiltration of ST2+FoxP3+Tregs in tumors, promoting the development of colon cancer. In addition, previous studies have suggested that IL-33 may affect Treg proliferation and immunosuppressive activity through type 2 innate lymphocytes, mast cells, and other unknown cells, thereby blocking the development of tumors. Nuclear respiratory factor 2-mediated targeting of the cross-talk between TICs can release IL-33. IL-33 activates the polarization of macrophages and the production of high-affinity IgE receptor-FcϵRIα, and FcϵRIα mediates TICs and promotes tumor invasion and drug resistance (79). These studies provide evidence for IL-33 as an inhibitor of tumor progression and a controversial understanding of the IL-33/ST2 signaling in tumor development.

#### 4.2.3 Regulatory role of IL-33 in metastasis

In addition to its regulatory role in proliferation regulation, IL-33 plays an important role in the control of tumor local invasion, migration, and metastasis. Metastasis is the most critical and destructive stage in the development of cancer and one of the reasons why cancer is difficult to cure completely. Among high-grade glioblastomas, glioblastoma multiforme (GBM) is the most common, aggressive, and lethal brain tumor, characterized by high proliferation and migration ability. IL-33 may play a crucial role in GBM progression by promoting GBM cell migration. In addition, in *in vitro* human glioma cell line experiments, IL-33 stimulated the secretion of matrix metallopeptidase 2 (MMP-2) and MMP-9 through the ST2-NF-κB pathway, thereby increasing the migration and invasion of tumor cells (86). In SW620 human colon cancer cells, IL-33 overexpression promoted tumor growth and migration *in vitro*, and tumor lung metastasis *in vivo*, while inhibiting IL-33 did the opposite (87). The metastatic microenvironment is the key to the spread and growth of tumor cells. Fibroblast-derived IL-33 alters the immune microenvironment and drives type 2 immunity to promote breast cancer metastasis (88). Serum levels of cytokines such as IL-17, IFN-γ, and TNF-α are increased, while the accumulation of activated NK and CD8^+^T cells in tumors is increased, suggesting that loss of IL-33 signaling promotes Th1/Th17 polarization of innate and acquired immune responses. IL-33 activates two pro-inflammatory factors, secreted IL-6 and IL-23, which lead to the expansion of Th17 cells and imbalance of Th17/Treg cells, ultimately leading to immune dysregulation, promoting the progression of inflammation and also promoting tumor growth in the tumor environment (89).

#### 4.2.4 IL-33 in tumor cell apoptosis

Intracellular proteolytic cleavage was initially thought to be the primary regulatory mode of IL-33 maturation. IL-1β and IL-18 need to be cleaved by caspase-1 to obtain biological activity (90), while flIL-33 can function as a cytokine. Caspases, as major intracellular proteases, were originally hypothesized to be involved in activating IL-33 because they play an important role in the cleavage/maturation of other IL-1 family cytokines. However, current literature suggests that caspases, as mediators of cell death, can inactivate IL-33 to suppress inappropriate inflammation during apoptosis. Recent studies have shown that IL-33 is not a substrate of Caspase-1 but can be degraded by pro-apoptotic Caspase-3 and Caspase-7, resulting in its inactivation (91). Therefore, the cleavage of caspases is a switch that inhibits the pro-inflammatory activity of IL-33 rather than a prerequisite for activating IL-33, ensuring immune tolerance during apoptosis by blocking IL-33 secretion. In addition, allergen proteinase-mediated cleavage of mouse and human FLIpl-33 leads to rapid degradation and a more stable form of mIL-33, respectively, which may depend on differences in amino acid sequence (92). Although studies on IL-33 hydrolysis have been conducted, other mechanisms of IL-33 proteolysis still need to be further explored. Proteases are also involved in regulating the biological activity of IL-33. For example, the calcium-dependent cysteine protease calpain, which is related with many cell death mechanisms such as apoptosis and necrosis, can also cut and activate IL-33 (93). During apoptosis, IL-33 is inactivated by the apoptotic proteases, blocking the immune response it activates upon apoptotic release. A study (94) has shown that although tumor-infiltrating Tregs have high early and late apoptosis rates, the rapid proliferation of Tregs in the TME is sufficient to overcome the apoptosis rate of tumor-infiltrating Tregs, resulting in the accumulation of Tregs in the TME. One aspect of the multiple differences in the role of IL-33 in some cancers may be related to differences in the ability of malignant cells to experience specific types of cell death. Owing to the dysregulation of mechanisms involved in cell death in patients with cancer, downstream IL-33 maturation may also be affected by alterations in caspase activity. IL-33/ST2 axis was involved in inhibiting apoptosis of gastric cancer cells by inducing the activation of ERK1/2, JNK, and p38 (95).

### 4.3 IL-33/ST2 and fibrosis diseases

Fibrotic disease is one of the leading causes of morbidity and death in humans, affects all tissues and organ systems, and may contribute to the progression of the disease. Repeated cell damage and chronic inflammation can cause excessive accumulation of ECM components, leading to the formation of permanent fibrotic scars. IL-33 has the dual role of cytokine and nuclear regulator. Similarly, the IL-33/ST2 signaling pathway plays a beneficial or harmful role in various fibrotic diseases, and IL-33 expression is closely related to the progression of various fibrosis. IL-33 promotes inflammation and fibrosis in most fibrotic diseases and reduces inflammation and fibrosis in others. IL-33 promotes an anti-inflammatory effect through ST2 interaction and a fibrotic effect through the ST2 membrane. For example, IL-33 has a protective effect in cardiac fibrosis and atherosclerosis; however, it can also induce lung, liver, intestinal, and skin fibrosis in an ST2-related manner.

#### 4.3.1 Pulmonary fibrosis

Many lung diseases are associated with the ultimate common pathway of pulmonary fibrosis. IL-33 can promote ST2-associated pulmonary fibrosis by inducing the alternating activation of mouse macrophages and innate lymphoid cells (96). IL-33 receptor-mediated inflammatory response and pulmonary fibrosis are mediated by TNF receptor-associated factor 6 and ubiquitin-specific protease 38 (97). Similarly, one study showed that IL-33 promotes pulmonary fibrosis by directly acting on two of the most important cell types during fibrosis: alveolar epithelial cells and (myo)fibroblasts. However, the significance of sST2 in pulmonary fibrosis is not fully understood. A study has proved that in the absence of ST2, early, long-term, unresolved inflammation will occur, and IL-33 is an important pro-fibrotic cytokine, which signals through ST2, mainly through the recruitment of IL-6-dependent replacement-activated macrophages and guided pulmonary fibrosis, thereby promoting the occurrence and progression of pulmonary fibrosis (98).

#### 4.3.2 Cardiac fibrosis

Chronic injury can activate myofibroblasts, leading to an imbalance of novel molecules associated with cardiac fibrosis and IL-33/ST2 (99). Physiological stretching induces the release of IL-33 by myofibroblasts, which then binds to ST2 on the cardiomyocyte membrane, facilitating cell integrity and survival. In chronic conditions, local and neighboring cells may increase the release of sST2, a decoy for IL-33 that blocks its binding to ST2L, thereby promoting fibrosis. Damaged cardiac cells release IL-33 alarmin protein under biomechanical forces to prevent local cardiomyocyte death and tissue loss (100). Studies have shown that IL-33 has anti-hypertrophic and anti-fibrotic effects on cardiomyocytes. Chronic injury leads to an imbalance in tissues associated with cardiac fibrosis owing to the IL-33/ST2 signaling pathway activation, potentially resulting in cardiac fibrosis formation. The regulation of this pathway is influenced by sST2 soluble isomers secreted by cardiac fibroblasts to sequester IL-33 from the extracellular space (101). When sST2 levels are elevated aberrantly as a decoy soluble receptor, it can block the IL-33/ST2 signaling pathway and promote fibrotic and apoptotic signal conduction. Additionally, Cheyenne et al. found a correlation between expression levels of factors in myocardial tissue related to the IL-33/ST2 axis with TGF-β 1 and connective tissue growth factor expression levels (101). ST2 can be used as a biomarker for cardiac stress and fibrosis. Its circulating levels are currently approved as an additional stratifying factor for patients with angina pectoris. ILC2 cells contribute to alleviating cardiac fibrosis related to IL-33 (102), suggesting that ILC2 cells may be a therapeutic target in treating cardiac fibrosis.

#### 4.3.3 Liver fibrosis

Liver fibrosis is caused by the gradual accumulation of ECM proteins, and during the progression of liver fibrosis, continuous inflammation activates the hepatic stellate cells (HSC), leading to their transformation into fibrotic and proliferating cell types. IL-33 released by HSC activates the positive tendency of ST2, thereby promoting the progression of liver fibrosis (103). Chronic inflammatory responses may result in fibrotic outcomes, such as chronic hepatitis, which causes fibrosis, characterized by persistent inflammatory infiltration and Th2-polarized immune activation. In contrast, although Th1 cytokines produce a rapid and intense inflammatory response, they rarely cause fibrosis. In patients with chronic liver disease, IL-33 acts as a chemotactic agent for Th2, inducing type 2 polarization of CD4^+^T cells, thereby promoting liver fibrosis (104), Th2 cytokines, such as IL-6, IL-4, and IL-13, promoting the formation of fibrosis (105). Notably, one study suggests that inhibiting IL-33 may halt the progression of non-alcoholic fatty liver disease to liver fibrosis and subsequently to hepatocellular carcinoma (HCC), highlighting its potential as a novel therapeutic target (106). In mouse and human fibrotic livers, IL-33 and ST2 mRNA are overexpressed, and the main source of IL-33 is HSC. In addition, studies have found that the IL-33/ST2 signaling causes the activation and accumulation of ILC2 cells in the liver, and the activated ILC2 cells produce IL-13, which causes the activation and differentiation of HSC through the IL-4Rα-recombinant signal transducer and activator of transcription-6 (STAT-6) transcription factor-related pathway (107). However, other studies have demonstrated the inhibitory effect of IL-33 on the fibrosis process, and IL-33 showed a significant protective effect in liver ischemia/reperfusion mouse models, alleviating liver injury and limiting inflammatory activity (108).

#### 4.3.4 Intestinal fibrosis

IL-33 plays a key role in maintaining normal intestinal homeostasis. Although one preliminary study (80) suggested a causative role for IL-33 in patients with inflammatory bowel disease (IBD), ST2s isoform mRNA in intestinal mucosa, is increased in patients with an active state of ulcerative colitis (UC) rather than Crohn’s disease (CD) and healthy controls, suggesting a potential role for IL-33/ST2 in wound or ulcer healing. Chronic inflammation usually leads to tissue fibrosis, especially CD, which often causes extensive local intestinal fibrosis (109). IL-33 is an effective inducer of Th2 immune response, and many studies have shown that IL-33 is also a crucial mediator involved in mucosal healing and epithelial recovery and repair. IL-33 is associated with areas of impaired barrier function and is essential for maintaining normal intestinal homeostasis. IL-33 induces the secretion of glial cell-derived neurotrophic factor family ligands (52). IL-33 plays a crucial role in intestinal epithelial barrier homeostasis via maintaining tight junctions and negatively regulating local inflammatory responses. In addition, IL-33 affects the enteric nervous system, inducing intestinal hyperkinesis to expel invading parasites from the gut, which is an important regulator of the microbiome. Although intestinal fibrosis has been extensively studied, whether IL-33 is a result of intestinal inflammation or an important irritant that promotes the inflammatory response remains unclear.

#### 4.3.5 Skin fibrosis

IL-33 plays a pivotal role in skin pathology by inducing an IL-13-dependent mechanism of cutaneous fibrosis that involves eosinophils and recombination-activated gene-related lymphocytes. Eosinophils are known contributors to tissue remodeling and fibrosis. Research has demonstrated that IL-33 promotes skin fibrosis via the ST2 receptor signaling pathway, and inhibiting the IL-33/ST2 axis can effectively suppress this fibrotic process (110). However, one study showed that deleting the IL-33 receptor ST2 resulted in an overall loss of IL-33 cytokine function, a significant increase in skin fibrosis, and a decrease in the proportion of Treg-to-Th2 cells in the diseased skin. ST2 loss results in significantly increased levels of Th2 cells and IL-13 expression in the skin and exacerbates fibrotic outcomes. Similar to these results, the IL-33/Treg axis prevents the development of skin fibrosis in chronic dermatitis models, suggesting that IL-33-TREg signaling is the primary mediator of IL-33 cytokine function in patients with skin fibrosis (111). Therefore, the Th2-to-Treg ratio provides a reproducible measure of skin fibrosis severity, and inducing the IL-33/Treg signaling may provide a novel treatment for tissue fibrosis in skin fibrosis.

### 4.4 IL-33/ST2 axis in autoimmune diseases

IL-33, as a type 2 alarm factor, can be released from epithelial, epidermal, and stromal cells through host invasion and escape mechanisms to maintain tissue homeostasis; however, it can also cause allergic inflammation sometimes. Studies have shown that the primary immune targets of IL-33 are constitutive ST2-expressing adaptive and innate tissue-resident immune cells (112). Autoimmune diseases are chronic and often fatal diseases caused by the immune system’s abnormal activation of its antigens, and there are typically 80 different types of diseases. Although the above studies suggest that IL-33 can regulate inflammation, the development of autoimmune diseases was not observed in mice lacking IL-33; therefore, further studies are needed to confirm the ability of nuclear IL-33 to regulate inflammation. Immune abnormalities are characterized by immune tolerance imbalances driven by autoreactive Th1/Th17 and B cells (113). There are two pathways of the immune response that are particularly relevant to IL-33. For example, IL-33 can activate innate immune cells such as DC and ILC2 (114). In addition, IL-33 can induce polarization of M2 macrophages (115), activate immunosuppressive cells (such as regulatory T and myeloid suppressor cells), and play an essential role in maintaining tissue homeostasis, inflammation, and tissue damage repair. Therefore, IL-33 together with ST2 play an important role as a cytokine in the progression of inflammation and autoimmune diseases. The dual role of IL-33 is also reflected in the immune system, which promotes autoimmunity and anti-autoimmunity. Therefore, targeting IL-33 and ST2 may be an ideal target for therapy in the future.

#### 4.4.1 IL-33/ST2 axis in rheumatoid arthritis

RA is a chronic autoimmune disease, the main cause of which is the abnormal immune system. The patient’s immune system mistakenly regards its normal joint tissue as a threat and attacks it, resulting in typical symptoms, including joint dysfunction, cartilage destruction, and debilitating pain (40). Cytokine-related immunity is essential in RA progression (116). The lymphocyte differentiation balance of RA is biased toward the Th1 subtype, and the number of cells with Th2 or Treg phenotypes is reduced (117). In patients with RA, the expression level of IL-33 was positively correlated with the phenotype, severity, and flexibility of RA, and the serum level of IL-33 was correlated with IgM and rheumatoid factor levels (118). Studies have shown that reducing the expression of ST2 prevents IL-33-induced neutrophil migration, which may be a new strategy for anti-TNF-α treatment of inflammation (119). Since the expression of IL-33 and ST2 is up-regulated in patients with RA and the expression level is related to the progression of the disease, further studies are needed on the mechanism of IL-33/ST2 in the disease.

#### 4.4.2 IL-33/ST2 axis in type 1 diabetes

T1D is a chronic autoimmune disease characterized by enhanced Th1 and suppressed Th2 responses, causing progressive destruction of the pancreatic insulin-producing cells (120). However, the IL-33 and ST2 expression levels are higher in the serum of patients with T1D than in that of the healthy controls (121). Such evidence indicated that IL-33 and ST2 expression levels are novel markers for T1D clinical cytokines and drug responsiveness. Several *in vitro* and mouse model studies have investigated the role of IL-33/ST2 in patients with T1D. For example, ST2 gene deficiency in a mouse model enhanced diabetes susceptibility and was associated with the increased level of glycosuria and infiltrating cells, the loss of β cells, and mRNA expression of TNF-α, IL-17, and IFN-γ in the pancreatic lymph nodes (122). In non-obese diabetic mice, IL-33 can reduce the infiltration of immune cells (including CD3^+^ T cells and MPO^+^ neutrophils) in islets, increase the expression of ST2^+^, recombinant GATA3^+^, Treg-related molecules, and slow down the destruction of beta cells in mice, ultimately inhibiting or delaying the progression of the disease (123). Therefore, the findings suggest that the IL-33/ST2 pathway may delay the destruction of beta cells by regulating the immune response of the body, which suggests that the IL-33/ST2 pathway may be a promising therapeutic target for preventing T1D.

#### 4.4.3 IL-33/ST2 axis in systemic lupus erythematosus

SLE is a severe systematic autoimmune disorder characterized by pro-inflammatory molecules and atypical production of autoantibodies. One study showed that serum levels of IL-33 were higher in SLE patients compared to healthy individuals (124), as well as an association between IL-33 rs1891385 and SLE (125). Another study indicated that serum IL-33 and sST2 levels were significantly higher in patients with SLE than in healthy controls. sST2 can be considered as a substitute marker of disease progression and complications of nephritis (126). In addition, the diversity of IL-33 gene expression can be used to predict SLE susceptibility. Therefore, targeting the IL-33/ST2 signaling pathway may be another strategy for SLE treatment.

#### 4.4.4 IL-33/ST2 axis in Graves’ disease and Hashimoto thyroiditis

The onset of GD is caused by hyperthyroidism, which is caused by receptor antibodies stimulating the thyroid gland, resulting in excessive secretion of thyroid hormone. HT is characterized by hypothyroidism and leads to the development of the condition owing to an attack on the thyroid gland by thyroglobulin-associated T-cell-mediated autoimmune responses. A recent study has shown that the IL-33/ST2 pathway is involved in the pathogenesis of autoimmune thyroid diseases. For example, in patients with GD and HT, IL-33 expression levels are higher than normal (127). The effect of the IL-33/ST2 pathway on immune diseases is closely related to susceptibility to autoimmune thyroid disease and some genetic variants of IL-33/ST2 and its related neighboring genes, which provides new ideas for existing therapeutic approaches in the future.

### 4.5 IL-33/ST2 axis in inflammatory diseases

The IL-33/ST2 signaling pathway influences the activation and function of various cell types. The IL-33/ST2 axis plays a crucial role in the development of multiple inflammatory diseases by inducing the expression of various pro-inflammatory cytokines and chemokines. For example, after type 2-mediated inflammation occurs, cells release IL-33 to mediate type 2 immunity. In several diseases, IL-33 induces mast cells to secrete pro-inflammatory cytokines that accelerate the development of inflammatory diseases. One study has shown that the level of IL-33 secreted in tumor tissues is positively correlated with the infiltration of mast cells, and studies have demonstrated that IL-33 can increase the number of mast cells in tumors through apoptosis (22). When suffering from external injury or invasion by bacteria and viruses, various immune cells secrete cytokines, including IL-33, which will mediate ILC2 cells to activate Th0 cells to differentiate into Th2 cells through the GATA3/STAT6 pathway and secrete inflammatory factors, including IL-4, IL-5, and IL-13, which will promote the occurrence of inflammation. Although further research is needed on the effect of IL-33/ST2 on inflammatory diseases, existing studies suggest that the IL-33/ST2 axis may contribute to the occurrence and progression of autoinflammatory diseases.

#### 4.5.1 IL-33/ST2 axis in IBD

IBD is a chronic disease with highly complex pathogenesis. CD and UC are different forms of IBD. Tregs’ involvement in IBD pathogenesis prevents the dysregulated inflammatory response to environmental stimuli. In addition, there are some pathologic and physiological similarities and clinical correlations between IBD and asthma and other non-pulmonary allergic diseases, such as mast cell activity and IgE involvement in disease development (128). Studies have proved that serum IL-33 levels are elevated in patients with IBD compared with healthy patients (129). In addition, serum sST2 levels were elevated in patients with IBD compared with healthy patients, and sST2 expression levels were positively correlated with the disease severity. In addition, ulcer-associated myofibroblasts and intestinal epithelial cells are important sources of mucosal IL-33 progression (130).

#### 4.5.2 IL-33/ST2 axis in sepsis

Sepsis is a systemic infectious disease, which refers to the systemic immune system imbalance caused by infection caused by bacteria, fungi, and viruses, which can lead to the dysfunction of multiple organs (131). Sepsis occurs when the host’s immune system overreacts and becomes dysfunctional in response to inflammation from infection (132). Several studies have shown that expression levels of IL-33 or sST2 are elevated in the serum of patients with sepsis, suggesting that the IL-33/ST2 axis is associated with sepsis progression. In contrast, exogenous IL-33 has a protective effect in mouse cecal ligation and puncture (CLP)-induced sepsis models. In a similar study, in the mouse model of sepsis, IL-33 administration enhanced neutrophil inflow to the site of infection and played a biological role, thereby effectively curing and reducing mortality in CLP-induced sepsis mice (133). IL-33 may support polymorphonuclear neutrophil-mediated bacterial clearance during the early stages of bacterial sepsis. IL-33/ST2 signaling may drive delayed immunosuppression in sepsis. In conclusion, IL-33/ST2 has a dual role in sepsis. In addition, in clinical data, sST2 levels are considered a marker for identifying patients with sepsis at an early stage.

#### 4.5.3 IL-33/ST2 axis in asthma

Asthma is a common chronic inflammatory airway disease characterized by wheezing or shortness of breath. Childhood-onset asthma is the most common form associated with chronic TH2-driven allergic inflammatory processes (134). IL-33 is also involved in asthma development. IL-33 is expressed in human lung airway epithelial basal cells, endothelial cells, and fibroblasts, which reflects the possible pathogenic role of IL-33 and ST2 in asthma. In genetic association studies, multiple scattered genetic signals in the gene locus of IL33 independently promote asthma sensitivity. A study (135) showed increased airway remodeling in children with severe asthma with fungal sensitization (SAFS). Although the mechanism of SAFS remains unclear, a recent SAFS mouse model demonstrated a relationship between the expression level of IL-33 and remodeling mediator MMP-9 (136). As a downstream target of IL-33, MMP-9 is a sensitive marker and mediator in airway remodeling activity (137) and is easier to detect than IL-33 as a marker in sputum and BAL samples (138). This suggests that MMP-9 can be used as a soluble biomarker to evaluate the efficacy of blocking IL-33 in patients with SAFS. In addition, blocking the IL-33/ST2 signaling pathway effectively inhibits the occurrence of asthma in asthmatic mice. A study (139) has demonstrated that IL-33 can regulate mast cell function in many ways after binding to ST2 on the surface of mast cells, and mast cells are key effector cells of allergic airway inflammation in patients with asthma. After exposure to the allergen, mast cells are activated by surface crosslinked IgE, degranulation occurs, and the release of bioactive mediators and IL-33 can enhance the ability of mast cells to release inflammatory mediators and degranulation. Interestingly, a study has demonstrated that some asthma patients can selectively produce natural anti-IL-33 autoantibodies in their serum (140). These antibodies can improve key inflammatory responses in asthma and may lead to improved lung function in allergic asthma, offering new insights and potential therapeutic approaches for the treatment of this condition.

#### 4.5.4 IL-33/ST2 axis in atopic dermatitis

Although the pathogenesis of AE is still not well understood, patients with AE are characterized by itching, barrier breakdown, and inflammation, including the production of type 2 cytokines. IL-33 is overexpressed in keratinocytes of patients with AE as an inflammatory cytokine. The mechanism of cytokines is manifested in patients with AE. The blood eosinophils of patients with AE increased, while IL-33 can stimulate various cells, including ILC2s, to produce type 2 cytokines, and IL-33-stimulated basophilic cells activate ILC2s through IL-4. IL-33 can promote itching and scratching behavior by inducing IL-31. Further, scratching the skin encourages keratinocytes to release IL-33, creating a vicious cycle. IL-33 also reduces the skin’s barrier function; however, barrier breakdown leads to skin exposure to allergens that promote IL-33 release (141). IL-33 down-regulates the expression of claudin-1 tight-linking protein in keratinocytes through the STAT3 signaling pathway and plays a role in the skin barrier (142). IL-33 can immediately act on the keratinocytes to reduce the skin barrier function of the epidermis and stratum corneum. In the skin with barrier dysfunction, keratinocytes are more likely to secrete alarm factors such as IL-33 (143), and the destruction of the barrier will lead to the continuous exposure of the skin to allergens, which may also lead to the occurrence of AE (144). These new findings could help in developing novel therapeutics targeting IL-33.

### 4.6 IL-33/ST2 axis and other diseases

Evidence that the IL-33/ST2 signaling is involved in the pathogenesis of various kidney injury-related diseases is increasing. For example, although studies suggesting that IL-33 may serve as a biomarker for early chronic kidney disease exist (145), some studies have suggested that the challenging measurement of serum IL-33 concentrations may lead to false-positive results, prompting further accurate studies of IL-33 expression levels in serum samples (146). Nevertheless, ST2 and IL-33 are critical in kidney disease progression, and further studies are needed. sST2 is a United States Food and Drug Administration-approved prognostic biomarker for predicting mortality in patients with chronic heart failure. Previous studies have shown that sST2 can be used to diagnose heart failure and to predict mortality after cardiac surgery for heart failure and coronary artery disease (147); however, a study has highlighted the protective role of IL-33 in patients with heart disease and reduced levels of sST2 as an inducer of IL-33 may prevent the formation of fibrosis. In this study, when IL-33/ST2 signaling was regulated with β-blockers, the increased expression level of the IL-33/ST2 signaling pathway was negatively correlated with the levels of markers of fibrosis and inflammation, indicating that IL-33 has a protective effect on the heart. However, further studies are needed to confirm that beta blockers mediate the IL-33/ST2 signaling pathway as therapeutic targets (148). IL-33 significantly increases Th2 cytokine levels in serum and lymph node cells, reduces Th1 (IFN-γ) levels, and promotes the progression of inflammation in cardiomyocytes. In addition, studies have shown that elevated ST2 expression levels can be used to predict the progression of myocardial fibrosis and heart failure in acute and chronic heart failure. As an indicator of myocardial fibrosis, ST2 plays a crucial role in evaluating the efficacy of drugs. In addition, studies have suggested that the IL-33/ST2 signaling pathway may be a key driver of mast cell-associated histaminergic pruritus (149). At the same time, a new study has demonstrated that the IL-33/ST2 axis has a protective effect against acute inflammation in a periodontitis model (150).

As the mechanism of the IL-33/ST2 signaling pathway in various diseases has been widely studied, the relationship between the IL-33/ST2 axis and ocular diseases has been paid more attention. A growing body of research has shown that the IL33/ST2 axis plays a key role in several eye diseases, including dry eye disease (DED), allergic eye disease, and visual neurotransmission disorders. IL-33 and ST2 proteins were elevated in conjunctiva impression cytology in patients with DED. In allergic eye disease, IL-33/ST2 mediates protoallergic reaction through the NF-κB signaling pathway, inducing the production of protoallergic cytokines and chemokines in corneal epithelial cells (151). In keratitis, IL-33 mRNA and protein levels were increased in the cornea of TH-2-responsive mice infected with Pseudomonas aeruginosa (152). In a mouse model of uveitis, up-regulating the IL-33/ST2 axis promoted the polarization of mouse macrophages and reduced the disease severity (153). In a vitreoretinal disease model of mice infected with damaged retinas by Toxoplasma gondii, elevated IL-33/ST2 expression levels up-regulated mRNA levels of trigger receptors expressed on TLR (154). In summary, IL-33/ST2 is promising in developing potential therapeutic strategies for eye diseases, and further research is needed.

## 5 Therapeutic strategies targeting the IL-33/ST2 pathway

Recently, with the increasing attention to the mechanism of action of the IL-33/ST2 pathway in different diseases, various antibody drugs related to the IL-33/ST2 pathway have been developed, and even anti-body drugs targeting IL-33 and ST2 have entered pre-clinical studies. Although pre-clinical studies of some drugs have been terminated or suspended owing to unsatisfactory efficacy and side effects, the importance of investigating therapeutic strategies targeting the IL-33/ST2 pathway remains undeniable. Antibiotic targeting IL-33 or ST3 have been considered in pre-clinical studies (**Table 1**), and the therapeutic effects in different areas provide insights for future research. In addition, some studies have also shown that small molecule substances and some natural compounds also show inhibitory effects on this pathway, and the collation of relevant studies on small molecule substances and natural compounds may provide new ideas for future therapeutic strategy research and drug development.

**Table 1:** Antibodies targeting IL-33 or ST2.

Name	Target	Diseases	Clinical stage	Clinical status	NCT
Astegolimab	ST2	Chronic obstructive pulmonary disease, novel coronavirus infection, Atopic dermatitis	Phase 2	Completed	NCT03615040
			Phase 3	Recruiting	NCT05595642
			Phase 3	Suspended	PACTR202305580698795
			Phase 3	Recruiting	NCT05878769
TQC2938	ST2	Severe asthma	Phase 1	Active, not recruiting	NCT06449859
			Phase 1	Recruiting	CTR20230916
9MW1911	ST2	Chronic obstructive pulmonary disease, asthma	Phase 1/2	Recruiting	NCT06175351
			Phase 1	Active, not recruiting	NCT05803902
			Phase 1/2	Recruiting	CTR20230380
			Phase 1	Completed	NCT05170243
			Phase 1	Completed	CTR20213300
			Phase 1	Completed	CTR20212590
PF-06817024	IL-33	Nasal disease, atopic dermatitis	Phase 1	Completed	NCT02743871
MT-2990	IL-33	Pain, seasonal allergic rhinitis, endometriosis	Phase 1	Recruiting	NCT06196905
			Phase 2	Completed	NCT03840993
			Phase 1	Completed	NCT03570957
			Phase 1	Completed	NCT03156738
Etokimab	IL-33	Atopic dermatitis	Phase 2	Completed	NCT03614923
			Phase 2	Completed	NCT03533751
			Phase 2	Completed	NCT03469934
			Phase 2	Unknown	EUCTR2017-000647-40-GB
			Phase 2	Completed	NCT02920021
			Phase 2	Not yet recruiting	EUCTR2016-002539-14-GB
			Phase 1	Completed	ACTRN12616000049471
PF-07264660	IL-33	Immune system diseases	Phase 2	Recruiting	NCT05995964
			Phase 2	Not yet recruiting	CTR20242718
			Phase 1	Completed	NCT05496738
Itepekimab	IL-33	Chronic obstructive pulmonary disease	Phase 3	Not yet recruiting	CTRI/2024/06/069046
			Phase 3	Not yet recruiting	CTR20241409
			Phase 3	Recruiting	NCT06208306
			Phase 1	Completed	NCT06114238
			Phase 3	Recruiting	CTRI/2022/12/048280
			Phase 2	Recruiting	NCT05326412
			Phase 3	Active, not recruiting	NCT04751487
			Phase 3	Active, not recruiting	NCT04701983
			Phase 2	Completed	NCT03546907
Tozorakimab	IL-33	Chronic obstructive pulmonary disease	Phase 3	Not yet recruiting	CTR20240891
			Phase 3	Not yet recruiting	CTR20232953
			Phase 3	Recruiting	NCT06040086
			Phase 3	Recruiting	NCT05742802
			Phase 3	Recruiting	CTR20221338
			Phase 3	Recruiting	NCT05166889
			Phase 3	Recruiting	NCT05158387
			Phase 2	Not yet recruiting	EUCTR2020-000571-20-DE
			Phase 1	Completed	NCT03096795
Melrilimab	IL-33	Asthma, atopic dermatitis	Phase 1	Completed	NCT04366349
			Phase 2	Terminated	NCT03393806
			Phase 2	Completed	NCT03207243
			Phase 1	Completed	NCT02345928

### 5.1 Antibodies targeting ST2

Research advancements have unveiled multiple ST2-related antibody drugs, some in pre-clinical stages. The IL-33/ST2 pathway is linked to asthma susceptibility, with inhaled allergens, respiratory viruses, and pollutants triggering IL-33 release and exacerbating asthma. Astegolimab, a full human IgG2 mAb targeting ST2, inhibited IL-33 signaling in a Phase 2b trial (NCT02918019) for severe asthma patients. A dose-ranging study (155) showed its efficacy in reducing AER, including in eosinophil-low asthma. Another study (156) assessed astegolimab’s safety and pharmacokinetics in severe AE (NCT03615040). Plasma IL-33 levels in atopic patients correlated with pro-inflammatory cytokines and cholesterol transport protein changes, impacting atherogenicity (157). In severe corona virus disease-19 (COVID-19) pneumonia, IL-33/ST2 pathway activation is associated with disease progression. However, a clinical study (158) randomizing 396 patients to astegolimab, efmarodocokin α, or placebo with standard treatment found no therapeutic benefit, despite biomarkers indicating pharmacological activity. Both treatments had similar adverse events, but did not shorten recovery time. No new safety signals have been observed, and adverse events were similar between the treatment groups. However, clinical results show that astegolimab or efmarodocokin alfa treatment does not shorten the recovery time of patients with severe COVID-19 pneumonia, which is contrary to the ideal results detected by biomarkers, and future studies need to be further conducted on this.

Furthermore, 9MW1911 is an innovative monoclonal antibody targeting ST2 for indications such as asthma, chronic obstructive pulmonary disease (COPD), and AE. Pre-clinical studies have shown that 9MW1911 has a high affinity with recombinant human ST2 at the molecular level and can effectively block IL-33 binding to ST2, inhibit the activation of NF-κB and MAPK signaling pathways, and inhibit the production of Th2 cytokines. The antibody drugs targeting ST2 or its ligand, IL-33, are currently unavailable worldwide. Similarly, 9MW1911 has completed the Phase I clinical dose climb. The study results show that it is safe and well tolerated. It is currently in the Phase Ib/IIa clinical initiation stage.

TQC2938 is a humanized IgG2 monoclonal antibody against ST2 (IL-33 receptor), belonging to Class 1 of therapeutic biologics. TQC2938 can specifically bind to human ST2, block its interaction with IL-33 ligand, block the ST2/IL-33 signaling pathway, reduce the production of downstream Th2 cytokines (such as IL-4, IL-5, and IL-13), reduce the level of eosinophils and other inflammatory cells, and reduce the occurrence of asthma, improving the quality of life of patients. Pre-clinical animal efficacy tests have shown that the candidate molecule can significantly improve disease symptoms.

### 5.2 Antibodies targeting IL-33

IL-33 antibodies, including PF-06817024, have been developed for pre-clinical trials. Following intravenous administration, PF-06817024 exhibited an average clearance rate of 0.058 mL/h/kg, a steady-state distribution volume of 56 mL/kg, and a terminal half-life of approximately 18 days in preclinical studies (159). This antibody specifically binds to IL-33 with high affinity, without significant interaction with other IL-1 family cytokines. By neutralizing IL-33, PF-06817024 inhibits the IL-33/ST2-mediated downstream signaling pathway, thereby blocking type 2 inflammatory responses. *In vitro* studies demonstrated its inhibitory effect on IL-33 activity in human mast cells and peripheral blood mononuclear cells. An *in vivo* toxicity study in cynophagus monkeys showed affinity for sudden IL-33, although the antibody does not cross-react with species other than non-human primates, complicating traditional antibody modeling. Based on cynomolgus monkey pharmacokinetics (PK), the estimated human PK half-life of PF-06817024 is 41 days. Clinical trials further confirmed the antibody’s safety and tolerability, with no dose-limiting toxicity observed. Overall, PF-06817024’s PK profile and safety data support its potential as a therapeutic option for allergic diseases.

As an IL-33 inhibitor, MT-2990 is currently under investigation for anti-neutrophil cytoplasmic antibody-associated vasculitis. Currently, six clinical studies on MT-2990 have been conducted, mainly on endometriosis, neutrophil cytoplasmic antibody-associated vasculitis, and ADs, and most clinical trials have been suspended or terminated. Only clinical studies against neutrophil cytoplasmic antibody-associated vasculitis are continuing in Japan. A Phase 2a trial (NCT03840993) evaluated the safety and efficacy of MT-2990 in adult patients with endometriosis. The current clinical study of the drug for endometriosis is currently in phase 2 but has been terminated or suspended.

Etokimab, also known as ANB020, is an IL-33 inhibitor and a monoclonal antibody drug, and the highest clinical phase study is in the third clinical phase. Seven clinical trials exist, most of which are in the completed stage, and 10 related patents are currently under study for respiratory diseases. Further research on cetuximab as a drug is necessary for the effect of cetuximab on inhibiting IL-33 was further confirmed by the reduction of eosinophils in the blood after treatment with cetuximab in humans who had lost IL-33 function (160).

A study (161) has shown that cetuximab affects IL-33 and inhibits neutrophil release mainly from two aspects. First, cetuximab inhibits *in vitro* IL-33-induced migration of neutrophils into skin interstitial fluid. Cetuximab can inhibit the neurosecretory IL-33 response of neutrophils to the skin interstitial fluid. Therefore, IL-33 is a skin chemical attractant related to neutrophils, and biotargeted therapy can effectively inhibit the migration of neutrophils *in vivo* and *in vitro*, thereby blocking the occurrence of inflammation.

PF-07264660 is a monoclonal antibody to IL-33 and inhibits other cytokines in the IL-1 family, such as IL-13 and IL-4. The main areas of research are diseases of the immune system, genetic diseases, deformities, and skin and musculoskeletal disorders. AE is a disease that is currently being studied. Two clinical trials of PF-07264660 are being conducted, which are currently undergoing a Phase 2 randomized, double-blind, placebo-controlled trial (NCT05995964) to evaluate the efficacy and safety of PF-07264660 in adults with moderate to severe AE (162).

Tozorakimab (also known as MEDI3506) is an anti-IL-33 monoclonal antibody that inhibits IL-33 reduction (IL-33red) and oxidation (IL-33ox). This is achieved primarily by stimulating the ST2 and advanced glycosylation end products/epidermal growth factor receptor signaling pathways and increasing epithelial cell migration and repair *in vitro* (163). Tozorakimab is a novel therapeutic drug with a dual mechanism of action, and 10 clinical trials on Tozorakimab exist. Blocking IL-33red and IL-33ox signaling can reduce inflammation and epithelial dysfunction in human diseases.

Melrilimab, also known as CNTO 7160, is a monoclonal antibody against the IL-33 receptor. The main treatment studied is asthma or airway disease, with five clinical trials currently underway. In healthy individuals and patients with asthma or AE, melrilimab forms a complex by binding to the extracellular domain of IL-33R and sIL-33R and is dose-dependent in inhibiting free sIL-33R/sST2. In addition, the increase in the total amount of SIL33R/sST2 and inhibition of IL-33-induced phosphorylation of p38 suggest that melrilimab’s effect on IL-33R/ST2 signaling can be sustained. Therefore, the clinical study of melrilimab in patients with asthma and AE was further supported (164).

### 5.3 Small molecules targeting IL-33/ST2 axis

Molecular-targeted drugs are usually very effective in treating allergic diseases and other immune disorders, and they also intervene in the key targets of the pathophysiological occurrence and development of malignant tumors. Molecular-targeted drugs are safer for short-term use than conventional therapies. In addition to blocking IL-33 and its receptor, small molecules specifically targeting the IL-33/ST2 axis are also important therapeutic means for allergic inflammatory diseases (165). In graft-versus-host disease (GVHD), sST2 can be used as a prognostic biomarker for disease progression (166). IL-33 is the only ligand that binds sST2 and ST2, and after allogeneic hematopoietic cell transplantation, patients with steroid-resistant GVHD experienced elevated sST2 levels, followed by non-relapse-related death.

1-(furan-2-methyl) pyrrolidine was developed as a small molecule ST2 inhibitor, and the structure-activity relationship of the compound against ST2/IL-33 binding was established. 1-(furan-2-methyl) pyrrolidine, as an ST2 inhibitor, releases IL-33 from sST2 and may regulate the T cell response to treat GVHD, thereby achieving therapeutic effects. In addition, the study found that in the mouse GVHD model, XY-52 (Compound 32) can effectively reduce the expression level of sST2, reduce the severity of GVHD, and improve the survival rate, and the differential intervention of ST2 antibody can also improve the survival rate of the GVHD mouse model. Therefore, small molecules capable of blocking ST2-IL-33 interactions may produce similar therapeutic effects to mitigate GVHD development, and specific drug studies for diseases where sST2 is a biomarker can be conducted. The study of small molecule ST2 inhibitors discovered through high-throughput screening and computational analysis found that the most potent compound, iST2-1, reduced plasma sST2 levels, alleviated disease symptoms, improved survival, and maintained graft-anti-leukemia activity. However, iST2-1 needs to be further optimized and studied before a treatment for sST2-mediated inflammatory diseases can be developed.

### 5.4 Natural products targeting IL-33 or ST2

With the continuous deepening of research on medicinal plants, the research on natural compounds in various diseases has also been improved (167). Natural compounds have become a major source of new drug development, such as the study of the effects of natural products on tumors-unresectable hepatocellular carcinoma (HCC) (168). With the continuous in-depth study of the IL-33/ST2 pathway, various natural compounds that may affect the IL-33/ST2 pathway have been studied, and related therapeutic compounds will be developed. Presently, five natural compounds have been shown to inhibit IL-33 or ST2 and have shown activity/therapeutic potential (**Table 2**).

**Table 2:** Natural products targeting IL-33.

Name	Structure	Mechanism	Disease
Kaurenoic acid	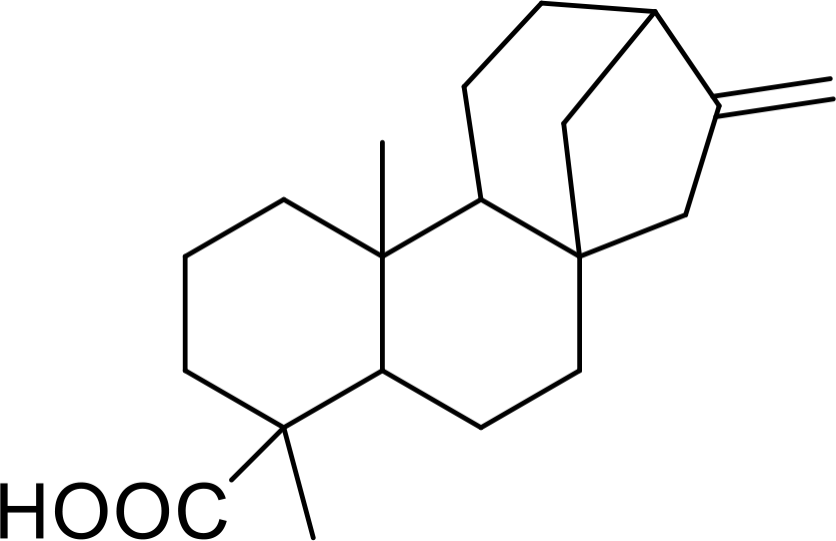	Decreased IL-33 production	Acetaminophen-induced liver necrosis
Vitexin	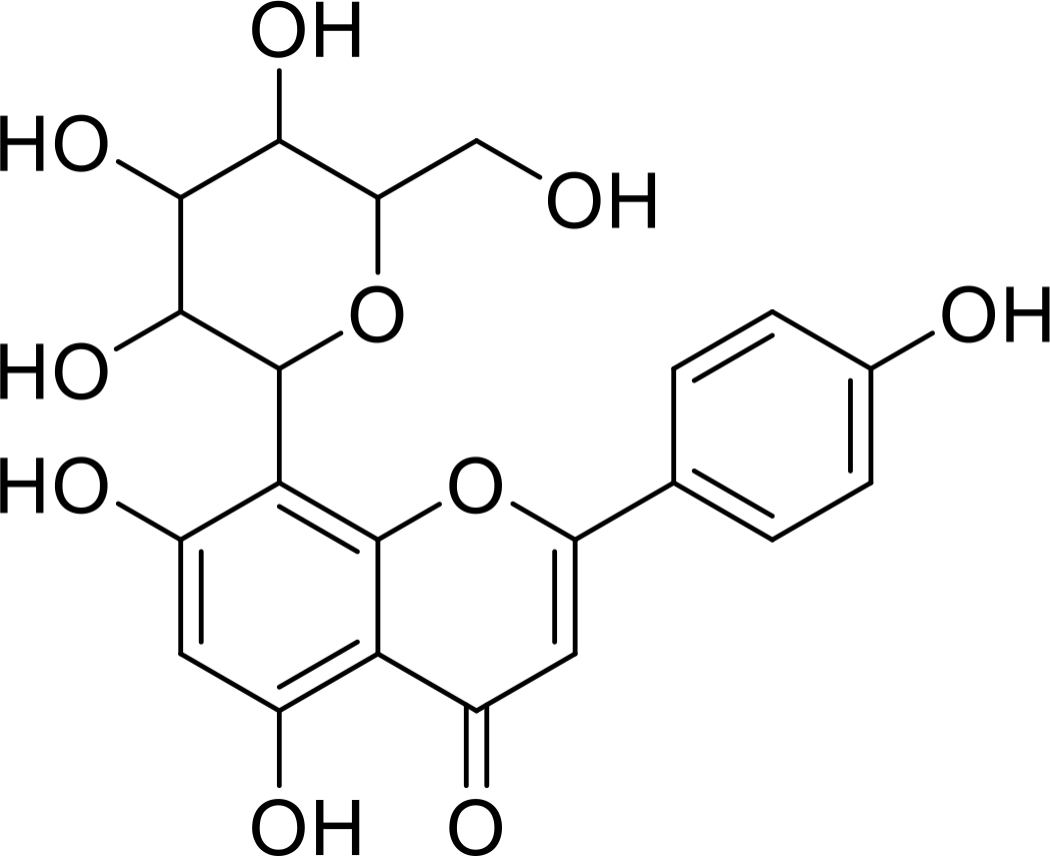	Decreased IL-33 production	Cardiovascular disease
Vanillic Acid	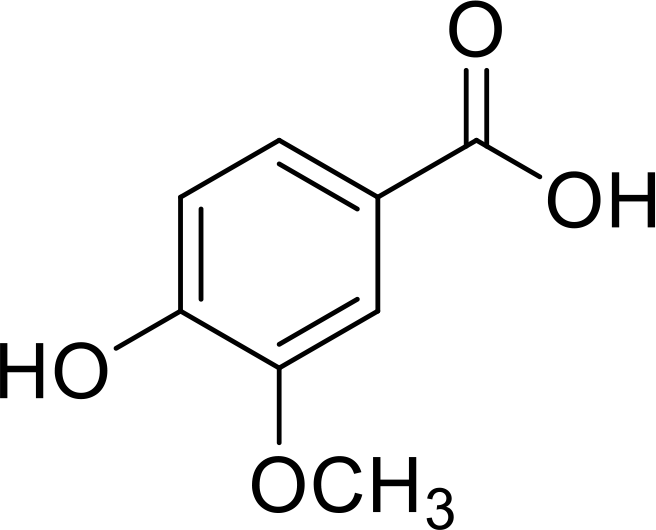	Decreased IL-33 production	Treatment with indomethacin
Quercetin	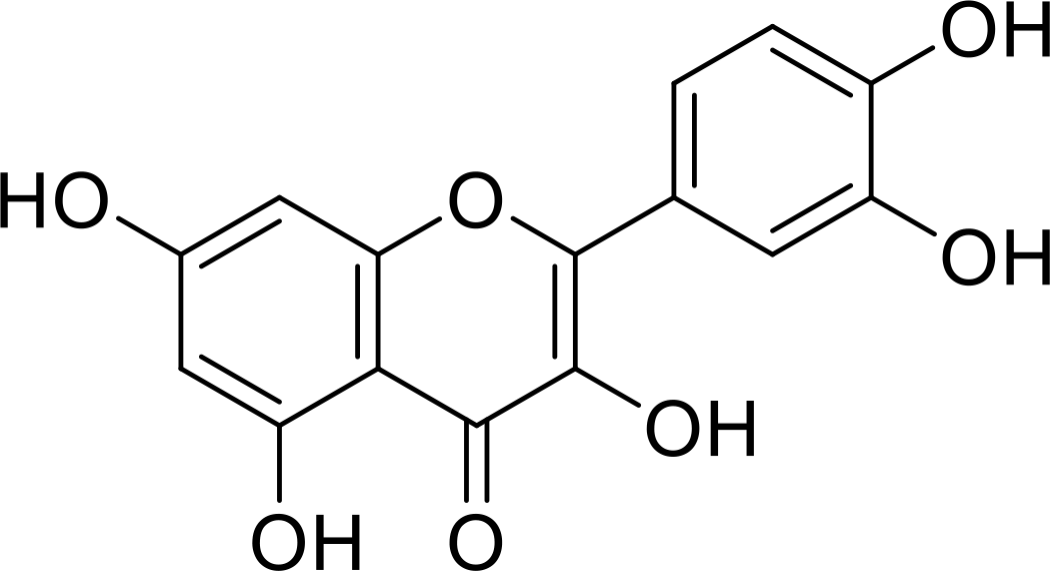	Decreased IL-33 production	Inflammatory and antioxidant
Matrine	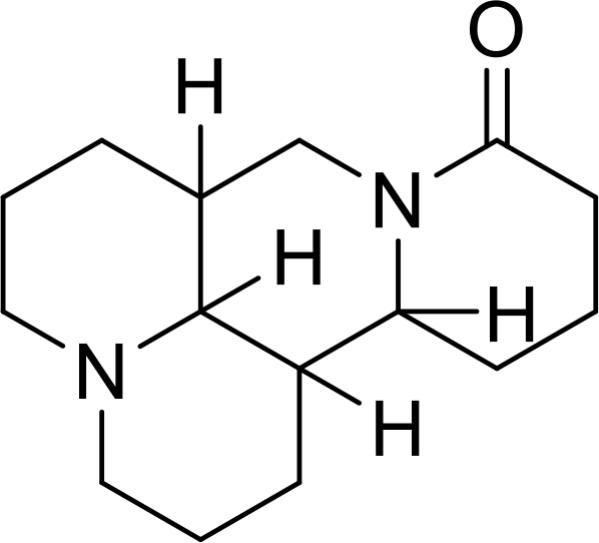	Decreased IL-33 production	Inflammatory diseases
Citric acid	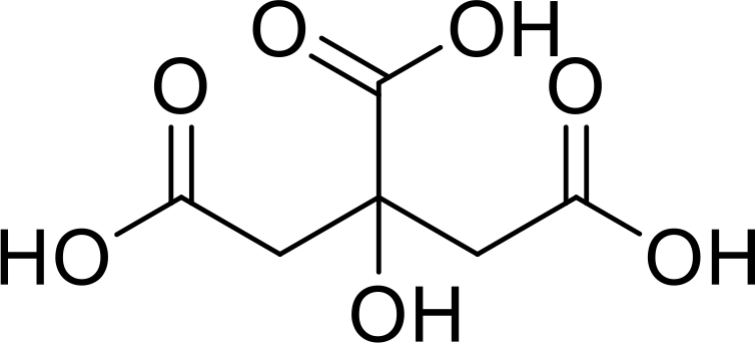	Decreased IL-33 production	Chronic spontaneous urticaria

Kynurenic acid (KA), a diterpene derived from Sphagneticola trilobate, reduces acetaminophen-induced liver necrosis and the expression levels of alanine aminotransferase and aspartate aminotransferase. KA reduces paracetamol-induced neutrophil and macrophage recruitment, oxidative stress, TNF-α, IL-33, and IL-1β production, and normalizes liver IL-10 levels, which is conducive to the study of KA against liver diseases (169). Vitexin is a natural flavonoid compound found in several medicinal plants and other plants (170). Vitexin has various pharmacological activities, including anti-inflammatory, anticancer, and antioxidant. Vitexin has an inhibitory effect on IL-33, a cytokine that also acts on various diseases. Vanillic acid is widely found in edible plants and fruits, which can inhibit NF-κB activation and has anti-inflammatory and antibacterial activities. In one study, indometacin was the control drug, and the results of Vanillic acid treatment showed that Vanillic acid could inhibit IL-33 production (171). Quercetin has various pharmacological activities and can be used as an anti-inflammatory and antioxidant flavonoid from several sources. For example, oral administration of Quercetin microcapsules can reduce neutrophil recruitment, accelerate damage repair, reduce edema, and decrease the production of inflammatory factors IL-1β and IL-33, thereby achieving a therapeutic effect. Elevated IL-1β and IL-33 levels in the inflamed mucosa are associated with UC pathogenesis (172). A study has shown that marine therapy regulates the inflammatory IL-33/ST2 axis by inhibiting the expression of IL-33 and ST2 mRNA, which is a new mechanism of marine (173). Citrate on desloratadine citrate disodium tablets reduces IL-33 levels. One study showed that IL-33 was treated by observing citrate on a desloratadine citrate disodium tablet (174). One study treated patients with chronic urticaria with citrate on desloratadine citrate disodium tablets and mizolastine for controls. After treatment, all patients showed a reduction in IL-33 levels. The results showed that citrate on desloratadine citrate disodium tablets had a more significant effect on chronic urticaria, and the level of IL-33 was significantly reduced after treatment (175).

## 6 Potential future developments and concluding remarks

While preclinical studies of antibodies and drugs targeting IL-33 and its receptor ST2 have shown promise, many clinical trials have been suspended or terminated due to side effects and limited efficacy. For instance, in a randomized Phase 2 trial involving 296 patients, itepekimab demonstrated improvements in condition and quality of life for severe asthma patients by blocking IL-33 expression compared to placebo (176). However, another Phase 2 trial with 343 patients found no benefit in reducing asthma exacerbations among smoking participants treated with itepekimab, and several side effects, including nasopharyngitis, bronchitis, headaches, and upper respiratory infections, were observed (177). In addition to side effects, treatment limitations have also been observed. A Phase 2a trial involving moderate to severe COPD patients treated with astegolimab showed improvements in health status, but astegolimab did not significantly reduce the rate of deterioration compared to placebo (178). Similarly, a Phase I trial involving 68 healthy subjects who received a single dose of intravenous CNTO7160 or placebo resulted in one case of severe cellulitis, raising concerns about the safety of CNTO7160. Furthermore, despite confirming target involvement, no significant clinical activity of CNTO7160 was observed in the final treatment of asthma patients (164), indicating that further research is needed to establish its therapeutic effect. Due to the side effects and limited treatment efficacy observed in these studies, additional research and exploration are necessary to improve the safety and effectiveness of antibodies and drugs targeting IL-33 and ST2 in clinical trials.

Current therapeutic strategies targeting the IL-33/ST2 signaling pathway primarily focus on blocking the expression of this pathway, such as reducing IL-33 release from immune cells, or blocking ST2 receptor signaling to achieve therapeutic effects. Additionally, research has summarized the role of the IL-33/ST2 signaling pathway in various diseases, which may lead to the development of relevant therapeutic strategies in the future. For example, in the heart, IL-33 has been shown to antagonize angiotensin II- and epinephrine-induced cardiomyocyte hypertrophy, and ST2-knockout mice exhibit increased ventricular hypertrophy, more severe fibrosis, and impaired survival. This suggests a possible protective effect of the IL-33/ST2 signaling pathway in the heart. However, no relevant drugs have yet been developed to verify this treatment strategy, and sST2 may serve as a biomarker to predict cardiovascular outcomes in the future.

In tumors, IL-33 plays a dual role, and future anti-tumor strategy research will require targeted studies based on different pathways in different tumors to develop effective treatment strategies. Notably, a recent review article (179) highlighted the direct association between the tumor suppressive effect of exogenous IL-33 and high immunogenic conventional dendritic cells (cDC1s), suggesting that promoting the immunogenicity of DCs with IL-33 may achieve a therapeutic effect. A new study has also presented a novel argument against the IL-33/ST2 pathway, revealing the molecular mechanism by which lineage-specific promoters reflect different expression patterns of ST2 in various T cell subsets. This provides a new research direction for IL-33 in the treatment of type I immunomodulatory inflammatory diseases and T cell-based tumor immunotherapy (180). Further drug development is urgently needed, and in addition to antibodies, small molecule substances and natural compounds that inhibit IL-33/ST2 have also been identified. We have compiled relevant small molecule substances and natural compounds that may be developed into new therapeutic drugs. The elucidation of the IL-33/ST2 pathway’s mechanism of action in various systems, including its role in pathogenesis and potential cures, provides a comprehensive framework for future drug development and treatment strategy research.
